# Toward Sustainable 3D-Printed Sensor: Green Fabrication of CNT-Enhanced PLA Nanocomposite via Solution Casting

**DOI:** 10.3390/ma17235782

**Published:** 2024-11-25

**Authors:** Javid Sharifi, Ghaus Rizvi, Haniyeh (Ramona) Fayazfar

**Affiliations:** 1 Eco-Friendly Circular Advanced Materials and Additive Manufacturing (ECAM) Lab, Department of Mechanical and Manufacturing Engineering, Ontario Tech University, Oshawa, ON L1G 0C5, Canada; javid.sharifi@ontariotechu.net; 2 Department of Mechanical and Manufacturing Engineering, Ontario Tech University, Oshawa, ON L1G 0C5, Canada; ghaus.rizvi@ontariotechu.ca

**Keywords:** fused deposition modeling, 3D printing, sustainable materials, nanocomposites, electrochemical sensors

## Abstract

The current study explores, for the first time, an eco-friendly solution casting method using a green solvent, ethyl acetate, to prepare feedstock/filaments from polylactic acid (PLA) biopolymer reinforced with carbon nanotubes (CNTs), followed by 3D printing and surface activation for biosensing applications. Comprehensive measurements of thermal, electrical, rheological, microstructural, and mechanical properties of developed feedstock and 3D-printed parts were performed and analyzed. Herein, adding 2 wt.% CNTs to the PLA matrix marked the electrical percolation, achieving conductivity of 8.3 × 10^−3^ S.m^−1^, thanks to the uniform distribution of CNTs within the PLA matrix facilitated by the solution casting method. Rheological assessments paralleled these findings; the addition of 2 wt.% CNTs transitioned the nanocomposite from liquid-like to a solid-like behavior with a percolated network structure, significantly elevating rheological properties compared to the composite with 1 wt.% CNTs. Mechanical evaluations of the printed samples revealed improvement in tensile strength and modulus compared to virgin PLA by a uniform distribution of 2 wt.% CNTs into PLA, with an increase of 14.5% and 10.3%, respectively. To further enhance the electrical conductivity and sensing capabilities of the developed samples, an electrochemical surface activation treatment was applied to as-printed nanocomposite samples. The field-emission scanning electron microscopy (FE-SEM) analysis confirmed that this surface activation effectively exposed the CNTs to the surface of 3D-printed parts by removing a thin layer of polymer from the surface, thereby optimizing the composite’s electroconductivity performance. The findings of this study underscore the potential of the proposed eco-friendly method in developing advanced 3D-printed bio-nanocomposites based on carbon nanotubes and biopolymers, using a green solution casting and cost-effective material extrusion 3D-printing method, for electrochemical-sensing applications.

## 1. Introduction

Biosensors are known as analytical devices designed to convert biochemical reactions into measurable electrical signals. These signals are directly proportional to the concentration of a particular substance, referred to as an analyte, within the reaction matrix [1]. Biosensors are an alternative to traditional clinical diagnostics systems, like magnetic resonance imaging (MRI) and computerized tomography (CT), presenting more feasible point-of-care diagnosis due to their selectivity, sensitivity, cost-effectiveness, and ease of manufacturing. Their application spans across a variety of sectors, including environmental monitoring and the medical industry [1,2,3,4,5]. However, contemporary fabrication methods have yet to address challenges inherent to biosensors. Conventional sensor manufacturing methods like photolithography, screen printing, coating/drop casting, and injection molding are limited by high processing times, complexity, and lack of scalability and flexibility, resulting in rigid sensors that are unsuitable for diverse applications. The conventional techniques further illustrate limitation towards fabricating complex geometries, produce high material waste, and require expensive materials and specialized equipment [6,7,8]. Additive manufacturing (AM), among other techniques that recently grabbed the attention of scientists and researchers in biosensor development, offers design flexibility, rapid fabrication, low material costs, and minimal waste, making it ideal for fabricating compact, complex, and efficient biosensing devices [9,10,11]. AM allows for intricate geometries and optimized electrode architectures, enhancing surface area and mass transport properties, which would lead to improved analyte diffusion, efficient electron transfer, and improved sensitivity, critical for electrochemical detection methods [8,12]. Among AM methods, fused deposition modeling (FDM) is predominant for its simplicity, affordability, and minimal waste production. FDM typically employs non-conductive thermoplastics filaments, like polylactic acid (PLA), which, despite its biodegradability and biocompatibility, has limited functionality in biomedical applications due to its insulating nature [12,13,14].

Integrating nanomaterials (such as graphene, carbon nanotubes (CNTs), etc.) into thermoplastic polymers effectively enhances their properties, like conductivity and mechanical strength. Polymer nanocomposites (PNCs) exhibit improved electrical, thermal, mechanical, optical, and antibacterial properties due to the synergistic effects between nanomaterials and polymer matrices. Such advancements have expanded PNC applications for next-generation nanodevices, like biosensors and electronics. Using sustainable bio-renewable polymers for PNCs offers an eco-friendly alternative to traditional petroleum-based polymers, contributing to reduced plastic consumption and a greener environment [14,15,16,17,18].

Reinforcing CNTs to biopolymer matrices in a nanocomposite configuration improves polymer properties, while preserving polymer biodegradability. The unique features of CNTs, including high aspect ratio, lightweight, and superior electrical and mechanical properties, make them ideal for augmenting polymers in biosensing applications. This includes facilitating electron transfer, enhancing sensitivity and selectivity, providing electrochemical stability, and ensuring biocompatibility [19]. However, challenges with CNT dispersion in the polymer matrix could restrain the electrical conductivity and electrochemical performance of the biosensor. Addressing these challenges involves selecting suitable mixing methods to achieve appropriate CNT dispersion within polymer, along with modifying CNT surfaces for optimal integration [20,21,22]. Achieving PNCs with a lower percolation threshold is also of interest for enhancing biosensor properties such as mechanical strength, electrical conductivity, and thermal stability. The low percolation threshold effectively offsets the manufacturing complexities and degradation of mechanical properties commonly associated with high-reinforcement concentrations. Additionally, it reduces reliance on expensive nanomaterials, addressing environmental concerns linked to the overuse of nanomaterials, thus establishing a balance between improved performance, cost-effectiveness, and environmental sustainability [23,24].

Melt mixing and melt extrusion blending are well-known approaches for incorporating CNTs into polymer matrices, as explored by Ivanov et al. [25] and Mora et al. [26] in their studies on FDM 3D-printed PLA:CNT composites. Ivanov et al. [25] observed a conductivity increase from about 8 × 10^−10^ to 2.1 × 10^−2^ S.m^−1^ by adding 0 to 6 wt.% CNTs to PLA. Mora et al. [26] illustrated a low percolation threshold at 0.23 vol.% CNT in FDM-printed composites, with a conductivity of around 5 × 10^−7^ S.m^−1^. Despite the effectiveness of melt mixing in these studies to prepare PLA:CNT feedstock for FDM printing, it could lead to potential CNT and polymer degradation due to high temperatures, impacting the composites properties for sensor applications. Alternatively, the solution casting method offers controlled CNT dispersion within polymer at lower temperatures, facilitates ease of operation, and achieves more effective dispersion of CNTs within the polymer matrix at lower concentrations of CNTs, in comparison to melt mixing [27,28]. The solution casting method, used by Kim et al. [29] to make PLA:CNT filaments using dichloromethane (DCM) as solvent for PLA, resulted in a conductivity of 1 × 10^−4^ S.m^−1^ by adding 1 wt.% CNTs compared to the baseline PLA matrix. Furthermore, the Young’s modulus of the composite exhibited an increase with the successive addition of CNTs. Notably, an enhancement of up to 50.34% in Young’s modulus was observed when 4 wt.% of CNTs was incorporated into PLA, in comparison to the baseline PLA matrix. Junpha et al. [30] achieved a conductivity of 110 S.m^−1^ using a 10 wt.% CNT concentration within PLA, using the solution casting process with chloroform as a PLA solvent, while the lower concentrations were not examined. However, the use of toxic solvents like DCM [29] and chloroform [30] in the solution mixing process in these studies poses health risks due to their toxicity and carcinogenic potential, raising sustainability concerns [31,32]. For example, plenty of the aforementioned solvents commonly used to dissolve PLA polymer are toxic and harmful, and their evaporation during dissolution of PLA and the subsequent drying of PNCs could lead to environmental pollution and pose health hazards. Therefore, exploring environmentally friendly or green solvents as alternatives is of both fundamental and practical importance for the preparation of PNC composites [33]. Moreover, among these studies, only Junpha et al. [30] explored the electrochemical-sensing capabilities of FDM-printed PLA:CNT samples towards potassium hexacyanoferrate (K_4_Fe(CN)_6_), hydrogen peroxide (H_2_O_2_), and nicotinamide adenine dinucleotide (NADH) at varying concentrations, to the best of authors’ knowledge. Particularly, a high CNT loading of 10 wt.% was used in this study, enabling the 3D-printed PLA:CNT electrodes to have an enhanced electrochemical detection performance. The 3D-printed electrodes illustrated detection limits of 9.8 μM and sensitivities of 12.3 μA/mM for potassium ferricyanide (K_4_Fe(CN)_6_), approximately 2.9 μM and 41.2 μA/mM for NADH, and a sensitivity of 22.6 μA/mM and a low detection limit of 5.3 μm for H_2_O_2_.

Therefore, a knowledge gap exists regarding how an appropriate eco-friendly solution to prepare CNT–biopolymer-based feedstock for material-extrusion 3D printing can enhance the dispersion of fillers within the polymer matrix, leading to improved conductivity and mechanical properties of 3D-printed parts. The uniform distribution of CNTs within the polymer matrix enhances the electrical conductivity of 3D-printed components with a lower amount of CNT content (decreased percolation threshold). To address this gap, in this study, for the first time, an eco-friendly solution casting method using green ethyl acetate solvent, was employed to prepare a PLA:CNT nanocomposite for an FDM 3D-printed working electrode in an electrochemical biosensor. This motive is based on the need to move away from traditional, toxic solvents in preparing CNT–polymeric nanocomposites that pose significant environmental and health risks. Ethyl acetate not only aligns with the principles of green chemistry but also proves to be a highly effective solvent for PLA [33,34]. Its adoption over hazardous solvents like chloroform and DCM represents a significant stride towards safer, more sustainable industrial and scientific practices.

After optimization of the PLA:CNT nanocomposite feedstock formulation, a low-cost desktop Filabot extruder was used to make 3D-printable filaments, and a desktop Prusa MK3S+ FDM printer was subsequently used to 3D print electrode samples. The thermal, rheological, morphological, and electrical conductivities of developed feedstocks and 3D-printed parts were examined fully with varying CNT concentrations. To further enhance the electrical conductivity of printed parts for electrochemical-sensing applications, an innovative electrochemical surface activation method was employed to remove a thin layer of polymer PLA from the surface and expose the embedded CNTs on the surface of samples. An electrochemical analysis was conducted on both activated and as-printed electrode samples, using 5 mM ferro/ferricyanide analyte as the electroactive probe. It was demonstrated that the ferro/ferricyanide redox couple agent, upon interacting with the activated electrodes, showed an oxidation peak with a current density of 17.6 μA.cm^−2^ compared to the as-printed electrode, which exhibited no noticeable current density peak. The successful preliminary results of this study, marked by enhanced mechanical properties, electrical conductivity, and electrochemical activity, demonstrated the possibilities of developing and 3D printing highly conductive bio-nanocomposites using a sustainable approach for the advancement of the next generation of nanodevices, like carbon-based polymeric nanocomposite biosensors with the lowest possible percolation threshold.

## 2. Materials and Methods

### 2.1. Materials

The Luminy LX175 PLA was supplied from Total Corbion (Total Energies Corbion, Gorinchem, The Netherlands) with a melt flow index (MFI) and a density (ρ) of 6 g/10 min (at 210 °C/2.16 kg) and 1.24 g/cm^3^, respectively. Carboxyl functionalized multiwalled carbon nanotubes (MWCNT-COOH) were sourced from Cheap Tubes Inc. (Grafton, VT, USA), with a purity of 99.9 wt.%, an outer diameter ranging from 10 to 20 nm, and a length of 10–30 µm. High-performance liquid chromatography (HPLC)-grade methanol and ethyl acetate were provided by Fisher Scientific, Canada.

### 2.2. Methods

#### 2.2.1. Polymeric Nanocomposite (PNC) Preparation

In this study, carboxyl-functionalized MWCNTs were used because they demonstrated better dispersion within the polymer matrix compared to non-functionalized MWCNTs. This improved dispersion facilitates a more effective CNT network throughout the polymer, allowing for the formation of a percolation threshold to be achieved at low CNT concentrations [35]. Thereof, MWCNTs-COOH were reinforced into a PLA polymeric matrix using a green phase-inversion process known as non-solvent-induced phase separation (NIPSs) [13]. To prepare polymeric nanocomposites, PLA pellets were first dried overnight at 70 °C in an oven to remove moisture content. Subsequently, the dried PLA pellets were dissolved in ethyl acetate at 80 °C for 4 h, maintaining a PLA-to-ethyl-acetate ratio of 1:10 (*w*/*v*) under magnetic stirring. Once the solution was cooled to ambient temperature, MWCNTs-COOH was added to it, and the solution was then stirred for another 4 h. This mixture was then subjected to an IKA^®^ T25 digital ULTRA-TYRRAX^®^ homogenizer equipped with an S 25 N–10 G dispersing tool, operating at 12,000 rpm for 30 min, at room temperature. Subsequently, the PLA:CNTs:EA mixture was added to methanol under magnetic stirring, facilitating the phase separation and resulting in the precipitation of the PLA:CNT nanocomposite. After filtration, the composite was rinsed thoroughly with methanol to eliminate residual ethyl acetate. The prepared nanocomposite feedstock was then dried overnight at 50 °C in an oven to ensure complete methanol evaporation. All composite formulations, including a code chart, were tabulated in Table 1.

#### 2.2.2. Filament Fabrication and FDM 3D Printing

All formulations detailed in Table 1 were extruded using a Filabot EX2 single-screw extruder (with the length-to-diameter (L/D) ratio of 12/1.5875 cm), featuring a 1.75 mm nozzle diameter, operating at a nozzle temperature of 152.5 ± 2.5 °C and 5 rpm to produce standard 1.75 ± 0.1 mm filaments suitable for FDM 3D printing. A desktop 3D printer, Prusa MK3S+, was used for 3D printing. The conversion of STL files to the compatible G-code format for the 3D printer was achieved using Cura Slicer software (UltiMaker Cura 5.8). Printing parameters were the same for all samples, with bed and nozzle temperatures of 60 °C and 200 °C, respectively, with a 0.4 mm nozzle diameter, 0.2 mm layer height, 100% infill density, and a printing speed of 10 mm/s. All samples were printed in a flat-on-bed orientation. Table 2 provides a summary of the printing parameters. The filament fabrication and printing parameters were carefully selected as part of our primary optimization process to enhance the overall quality of the final printed samples.

## 3. Characterization Tests

### 3.1. Attenuated Total Reflection–Fourier-Transform Infrared Spectroscopy (ATR-FTIR)

FTIR spectroscopy of formulations from Table 1 was carried out using a PerkinElmer Spectrum 100 FT-IR Spectrometer equipped with horizontal attenuated total reflectance (HATR) accessory (Waltham, MA, USA). No additional preparation was required for the formulations. Infrared spectra were captured in absorption mode, covering a wavenumber range from 600 to 4000 cm^−1^, with a 4 cm^−1^ resolution, averaging 16 scans.

### 3.2. Thermal Analysis

All filaments from Table 1 formulations underwent thermal analysis using a differential scanning calorimetry (DSC) Q20 instrument (TA Instruments, New Castle, DE, USA) in a nitrogen atmosphere, at a flow rate of 50 mL/min. Roughly 5 mg of each ground sample was placed in an aluminum T_zero_ pan for DSC analysis. The samples were heated at 10 °C/min to 200 °C, maintained for three minutes, cooled to 30 °C at the same nitrogen flow rate, and again held for three minutes for thermal equilibrium. This process was followed by a second heating cycle up to 200 °C at 10 °C/min. For this study, the melting temperature (T_m_), cold crystallization temperature (T_CC_), and glass transition temperature (T_g_) were noted for both heating cycles. The second cycle was specifically analyzed to eliminate any influence from the processing thermal history. The crystallinity percentage of PLA (%X_CrPLA_) was determined using Equation (1), with the $H_{mPLA}^{0}$ value set at 93.7 J/g (representing the heat of fusion for 100% crystalline PLA), and values H_CCPLA_ and H_mPLA_ for cold crystallization and melting enthalpy of PLA, respectively [36].

(1) $$\%X_{\mathrm{CrPLA}}=(\frac{H_{mPLA}-H_{CCPLA}}{H_{mPLA}^{0}\times \left(PLA\;wt.\%\right)})\times 100$$

Contrarily, thermal stabilities of all filaments were investigated using thermogravimetric analysis (TGA), with a TGA Q50 instrument (TA Instruments, New Castle, DE, USA). For each test, an average of 3.5 mg of sample was heated from 30 to 600 °C at 10 °C/min in a nitrogen atmosphere, with a nitrogen flow rate of 40 mL/min.

It is worth noting that DSC and TGA analyses were each conducted three times on all filaments listed in Table 1 to assess the repeatability of the thermal characterization. The results demonstrated high consistency across all tests, with similar graphs obtained for each analysis.

### 3.3. Rheological Characteristics

For the rheological study of all formulations in Table 1, ASTM D4440 standard was employed while using a Discovery HR-2 Rheometer (TA Instruments, New Castle, DE, USA) with a sample height and radius of 2 and 12.5 mm, respectively. An oscillation frequency sweep, in angular frequency, from 0.1 to 100 rad/s at 190 °C, was performed for all 3D-printed samples, with a strain of 0.5% and a plate gap of 1.0 mm. Following the frequency sweep, the modified Cross model was employed to obtain the zero-shear viscosity (Equation (2)) for all formulations, where η is the viscosity, η_0_ is the zero viscosity, γ is the shear rate, τ_0_ is the yield stress, and m is the consistency index [37].

(2) $$\eta =\left(\frac{\eta_{0}}{\left(1+{\left(\tau_{0}\gamma \right)}^{m}\right)}\right)\;$$

### 3.4. Electrical Characteristics

Three cylindrical specimens of CNT-reinforced polymeric composites, each with different weight percentages of CNTs, were FDM 3D-printed. Each specimen had a diameter of 20 mm and a thickness of 0.3 mm. To moderate contact resistance, a uniform layer of conductive silver ink (M.E. Taylor Engineering Inc., Rockville, MD, USA) was applied to the grip areas on the surface of each specimen. The electrical conductivity of the specimens was subsequently evaluated using a Keysight E4990A Impedance Analyzer.

### 3.5. Mechanical Properties

Tensile tests were conducted up to the point of rupture on five replicates of each dog bone-shaped sample, as per ASTM D 638-14 Type V specifications [38]. A Universal Testing Machine (Instron^®^ 3400 series, Norwood, MA, USA) was used with a crosshead speed of 1 mm/min. Dog bone-shaped Type V samples were FDM 3D-printed based on the formulations detailed in Table 1, using the printing parameters listed in Table 2. Following the tests, properties such as the tensile modulus (E), tensile strength (σ_max_), elongation at break (ε%), toughness, and resilience were assessed.

### 3.6. Morphology and Electrochemical Performance

The surface morphology of the PNCs was thoroughly analyzed using a Schottky SU7000 Field-Emission Scanning Electron Microscopy (FE-SEM) with ultra-high resolution, with no need for sputter coating.

An electrochemical surface-activation method was utilized to selectively dissolve the insulating PLA layer from the electrode’s surface, thereby exposing the conductive CNTs on the surface, using a Gamry Reference 600 potentiostat/galvanostat controlled by Gamry framework. To this end, working electrode samples (sensors) were printed with an active surface area of 0.7 cm^2^ and analyzed using cyclic voltammetry (CV) in a 0.5 M sodium hydroxide (NaOH) solution (20 mL), under atmospheric pressure and at room temperature. The NaOH solution was selected for its saponification properties, effectively dissolving PLA and thereby enhancing electrode performance by exposing a higher load of CNTs on the surface [39]. To ensure the effective removal of PLA from the electrode surfaces, a potential window ranging from −1.0 V to +1.4 V was employed over 250 cycles, with respect to an Ag/AgCl reference electrode at a scan rate of 50 mV/s.

After the surface activation, the electrochemical performance of the FDM 3D-printed specimens was precisely evaluated using CV to detect a 5 mM [Fe(CN)_6_]^4−/3−^ redox couple agent (20 mL at atmospheric pressure and room temperature). This study aimed to highlight the effectiveness of the electrochemical activation technique in augmenting the performance of FDM 3D-printed parts for sensing applications.

## 4. Results and Discussion

### 4.1. FTIR Spectroscopy

**Figure 1:**
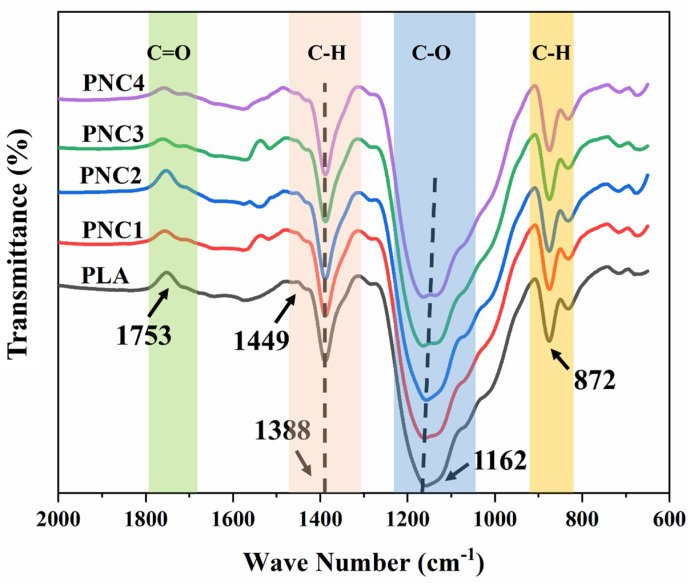
FTIR-ATR spectra of PLA, PNC1, PNC2, PNC3, and PNC4.

Figure 1 illustrates the Fourier-transform infrared (FT-IR) spectroscopy analysis of both Virgin PLA and PNCs. Virgin PLA displays a characteristic absorption band at 1449 cm^−1^ for CH_3_ stretching and a C-H deformation vibration at 1388 cm^−1^ [36]. The PNCs’ FTIR spectra reveal minor spectral alterations after the CNT incorporation, particularly a slight shift and sharpening of bands in the 1300–1450 cm^−1^ range. This suggests molecular interactions between CNTs and polymer chains, possibly arising from the PLA helical structure that forms a coiling polymer film around CNTs [40]. The band at 1753 cm^−1^ corresponds to the C=O stretching of carbonyl groups from COOH on the functionalized MWCNTs. The slight shift at this band suggests the successful incorporation of functionalized MWCNTs, providing additional interaction sites within the CNTs and PLA polymeric matrix. Additionally, the typical C-O stretching vibration from PLA, originally at 1162 cm^−1^, shifted to a lower wavenumber in the PNC samples. This signifies a potential adhesion between CNTs and the PLA matrix, likely due to interactions between the hydroxyl groups of PLA and hydroxyl on the CNT surface, facilitated through solution mixing [35]. Overall, the FTIR spectra of PNCs exhibit the characteristic vibration bands of both CNTs and PLA, confirming their integration within the CNTs-PLA matrix.

### 4.2. Thermal Analysis

**Figure 2:**
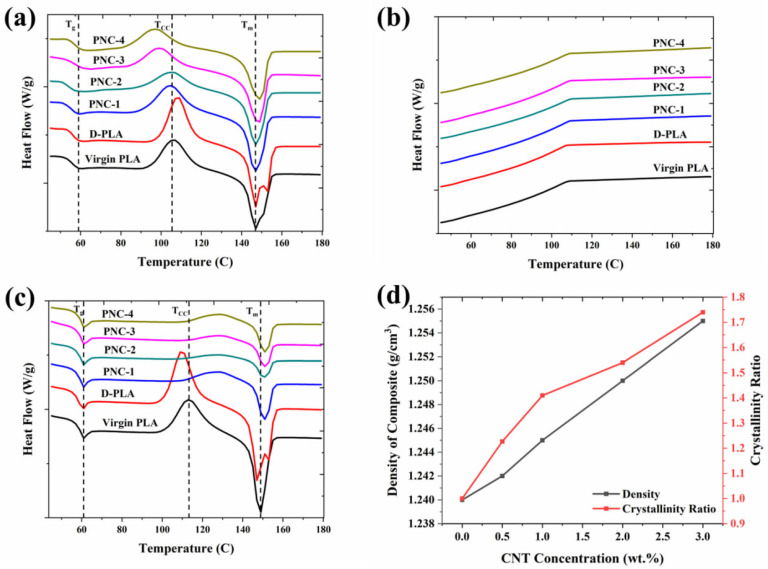
Differential scanning calorimetry of PNC composites: (**a**) first heating scan, (**b**) first cooling scan, (**c**) second heating scan, and (**d**) influence of CNT concentrations on density and crystallinity ratio (%X_CrPLA_/%X_CrPNC_) of PNCs.

Our DSC analysis revealed distinct thermal behaviors in PLA and D-PLA. PLA exhibited a single melting point without a clear crystallization temperature during cooling, suggesting incomplete crystal formation (Figure 2a,b). Conversely, D-PLA showed two melting points at 147 °C and 153 °C, likely due to different crystalline phases in PLA altered by ethyl acetate dissolution (Figure 2a). Rapid cooling of D-PLA resulted in imperfect crystal formation, with less stable crystals that are melting at lower temperatures during heating, followed by more stable crystals formed during recrystallization that are melting at higher temperatures [41].

DSC curves were also evaluated to investigate the effect of CNTs’ incorporation on the crystallization properties of PNCs (Figure 2a–c). In general, CNTs are known to serve as effective nucleating agents that, upon dispersion of CNTs in PNCs, enhance the crystallization rate of PLA [42,43]. During the first heating scan, a noticeable shift of the T_CC_ to a lower temperature relative to virgin PLA was observed, which could be attributed to the nucleating effect of the well-dispersed CNTs (Figure 2a). Moreover, there was a successive increase in the %X_CrPLA_ with the increasing CNT concentration, suggesting that CNTs act as nucleating agents in PLA, facilitating the crystallization process (Table 3) [22]. Subsequently, the PNC samples were subjected to a cooling scan, where no obvious crystallization peak was observed (Figure 2b).

Upon the second heating scan, the %X_CrPLA_ was successively increased with an increasing concentration of CNTs. However, in contrast to the first heating scan, the T_CC_ exhibited a positive temperature shift (Figure 2c). Within this context, it was observed that cold crystallization of PNCs consistently occurs at a faster rate (lower temperature) during the first heating scan (Figure 2a), while it happens at a slower rate (higher temperature) during the second heating scan (Figure 2c), in comparison to virgin PLA. More precisely, during the first heating scan, the PLA would melt, erasing the thermal history of the sample. The subsequent cooling scan promotes a more ordered crystal structure; therefore, the more ordered crystal structure necessitates greater energy and, subsequently, a higher temperature to initiate cold crystallization during the second heating scan [43,44].

Additionally, the T_g_ and T_m_ values in PNCs were found to be either equal or varied by only a difference of 2 °C compared to virgin PLA, across both the first and second heating scans (as illustrated in Table 3). This observation suggests that the incorporation of CNT filler has a minimal impact on the mobility of the polymer chains, as evidenced by the minimal impact of CNT filler on T_g_ and T_m_. Thus, the observed changes in cold crystallization (T_CC_) could primarily be attributed to variations in the nucleation effect of CNTs rather than the change in mobility of polymer chains [43].

To further validate the DSC findings, the density and crystallinity (from the second heating cycle) of D-PLA and PNCs were measured to assess variations in PLA crystallinity (as illustrated in Figure 2d). With the increasing CNT concentration, the PLA density exhibited a linear rise, moving from 1.24 g/cm^3^ to 1.255 g/cm^3^ (Figure 2d). Concurrently, the crystallinity ratio also increased in accordance with the CNT concentration, aligning with the DSC’s tabulated results (Table 3).

**Figure 3:**
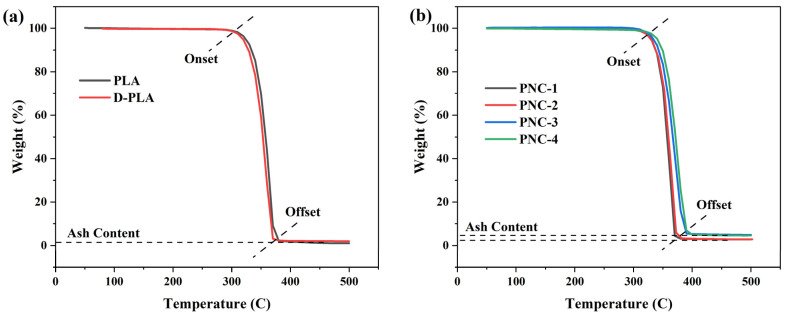
TGA thermograms of (**a**) virgin PLA and D-PLA, and (**b**) PNCs.

According to the thermogravimetric analysis (TGA) presented in Figure 3, a single-step degradation process is observed across all formulations. The onset degradation temperature of PLA is around 300 °C, which is primarily attributed to the loss of end groups (esters) from the main chain, culminating in complete decomposition at 380 °C [36]. In contrast, D-PLA reveals a comparable behavior, with an onset degradation temperature identical to PLA at 320 °C, but a decreased offset at 370 °C. This slight variation could be ascribed to the thermal history of D-PLA and the solution-mixing and filament-making process, which also influenced the crystallinity and structure of PLA. Furthermore, the incorporation of CNTs into the PLA matrix positively influenced its thermal stability. This is evident from the 6.67% shift in the onset degradation temperature, moving from 300 °C to 320 °C across all PNCs. Such enhanced thermal stability could likely be attributed to the superior thermal characteristics of CNTs, combined with the strong interfacial interaction between CNTs and the polymer matrix. Moreover, the ash content at 500 °C increased from roughly 1.5 wt.% in PLA to 4.6 wt.% in PNC-4, highlighting the presence and stability of CNTs even at 500 °C [45].

### 4.3. Rheology Characteristics of FDM 3D-Printed PNCs

**Figure 4:**
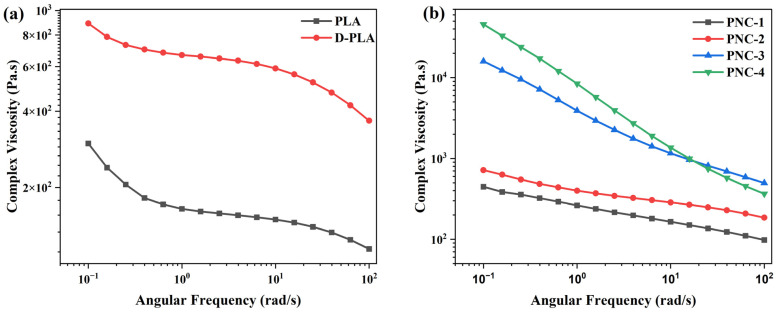
Complex viscosity of (**a**) PLA and D-PLA, and (**b**) PNCs.

The rheological properties of FDM 3D-printed samples, as detailed in Table 1, were examined and are depicted in Figure 4, while the resulting zero-shear viscosity for each specimen was tabulated and is presented in Table 4. PLA exhibited typical non-Newtonian shear-thinning behavior, with decreasing complex viscosity at higher angular frequencies [46]. D-PLA exhibited a similar behavior but with increased viscosity, potentially due to alterations in the polymer structure and crystallinity from dissolution and solidification processes (Figure 4a). By contrast, PNC samples displayed significantly higher viscosity compared to PLA, particularly in PNC-3 and PNC-4, highlighting the reinforcing effect of CNTs (Figure 4b). This increase in viscosity with the rising CNT concentration, noted from PNC-1 to PNC-4, can be attributed to the high aspect ratio of CNTs and their capacity to form interconnected networks within the PLA matrix. Notably, the rheological distinction observed between PNC-2 and PNC-3 suggests the occurrence of CNT percolation. At a 2 wt.% concentration of CNT within PLA (PNC-3), CNTs form a continuous path throughout the PLA matrix, thereby enhancing properties such as electrical conductivity and complex viscosity [13,47]. PNC-3 exhibited significantly steeper slopes and higher complex viscosity compared to PNC-2, implying that as the concentration of CNTs increases, the interaction between CNTs and PLA enhances. Consequently, the composite gradually approaches the percolation threshold (PNC-3, 2 wt.%), leading to a pronounced increase in complex viscosity.

**Figure 5:**
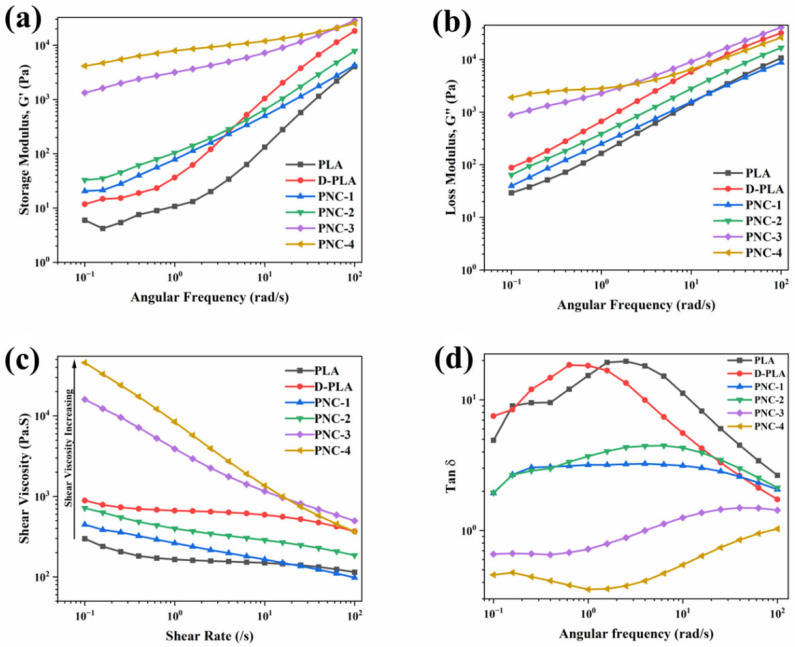
(**a**) Storage modulus, (**b**) loss modulus, (**c**) shear viscosity (Pa.S), and (**d**) Tan δ for different samples.

Figure 5a,b illustrate the storage (G’, representing the elastic part of material) and loss modulus (G”, representing the viscous part of material) of PNC composites, PLA, and D-PLA. For both PLA and D-PLA, there is a consistent rise in the G’ and G” moduli as the angular frequency increases. Particularly, D-PLA demonstrates a marginally higher modulus than PLA, potentially due to the alteration in PLA’s structure during the dissolution and solidification process. Furthermore, the increase in storage and loss modulus with the increased CNT concentration underscores CNTs’ reinforcing capability (Figure 5a,b). The reinforcing effect is further evident in shear viscosity (shown in Figure 5c), where the CNT concentration amplifies the inherent shear-thinning behavior of PLA [46,47].

Specifically, for most of the PNC samples, the loss modulus consistently remains higher than the storage modulus across all frequencies. However, a lower loss modulus was observed for PNC-3 at lower frequencies that surpasses the storage modulus at higher frequencies. In contrast, PNC-4 maintains a superior storage modulus throughout all frequencies. For both the storage and loss modulus, as the CNT concentration surpasses 1 wt.%, the modulus curve slopes for the PNCs experience a notable alteration. G’ remains nearly frequency-independent, indicating a pseudo-solid-like CNTs network within the polymer matrix. The transition from liquid-like to solid-like viscoelastic behavior in the lower frequency range, particularly observed between PNC-2 and PNC-3, highlights the considerable restriction of long-range motion in polymer chains. This is attributed to the influence of the CNT network within the composite. Furthermore, the frequency-dependent behavior of G” also shows the same tendency as G’ [47].

Figure 5d presents the variation in the loss tangent (Tan δ) across different CNT concentrations, a parameter crucial for assessing material viscoelasticity. Tan δ, more sensitive to relaxation changes than storage and loss modulus, displayed a distinct response to varying CNT concentrations. For PNC-1 and PNC-2, Tan δ decreased with increasing frequency, typical of viscoelastic liquids. However, PNC-3 and PNC-4 showed a moderate Tan δ increase with frequency, indicating a predominantly elastic response. This shift implies that, beyond a specific CNT concentration, likely between 1 and 2 wt.%, Tan δ becomes frequency-independent, marking the formation of a CNT percolation network [47].

### 4.4. Electrical Conductivity of FDM 3D-Printed PNCs

**Figure 6:**
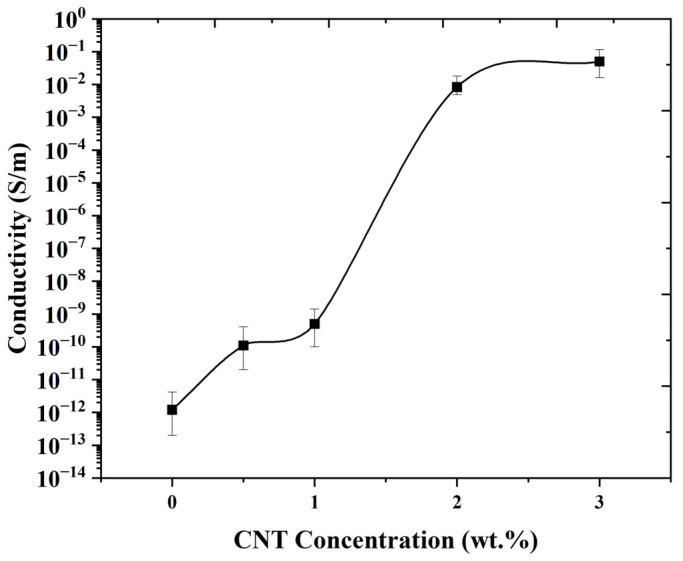
Electrical conductivity of FDM 3D-printed PNCs. (Standard deviations of ±2.1 × 10^−12^, ±1.96 × 10^−10^, ±6.5 × 10^−10^, ±6.5 × 10^−3^, and ±5.3 × 10^−2^ for 0, 0.5, 1, 2, and 3 wt.% of CNTs concentration, respectively).

Figure 6 illustrates that the electrical conductivity of FDM 3D-printed PNCs significantly increases with the addition of CNTs. The increased conductivity in PNCs is attributed to the inherent electrical properties of CNTs. Once integrated into the polymer matrix, CNTs form a conductive network that enhances electron transfer. CNTs’ high aspect ratio and surface area are also critical for establishing percolation pathways that boost conductivity, even at low CNT concentrations [21,22,23].

A notable increase in conductivity, from 5 × 10^−10^ S.m^−1^ at 1 wt.% CNTs to 0.0083 S.m^−1^ at 2 wt.%, indicates the onset of percolation around 1 wt.% CNTs. Introducing just 0.5 wt.% CNTs shifted conductivity from 1 × 10^−12^ S.m^−1^ (in virgin PLA) to 1 × 10^−10^ S.m^−1^, with minimal changes up to 1 wt.% CNTs, beyond which, a clear percolation effect is observed. The conductivity values of 0.0083 and 0.05 S.m^−1^ for 2 and 3 wt.% CNTs, respectively, underscore the formation of an interconnected conductive network of CNTs within the PLA matrix. After reaching the percolation threshold at around a 2 wt.% CNT concentration, where a notable increase in conductivity was illustrated, further increases in CNT content (e.g., 3 wt.%) demonstrate minimal changes. This plateau effect is attributed to the formation of a continuous conductive network within the polymer matrix. Beyond this point, additional CNTs tend to aggregate rather than form new conductive pathways, leading to saturation in electrical performance. This behavior aligns with percolation theory, which states that, beyond the critical concentration, an additional filler has little effect on electrical properties, as the three-dimensional conductive network is fully established at the percolation threshold [48,49].

### 4.5. Mechanical Characteristics of FDM 3D-Printed Specimens

**Figure 7:**
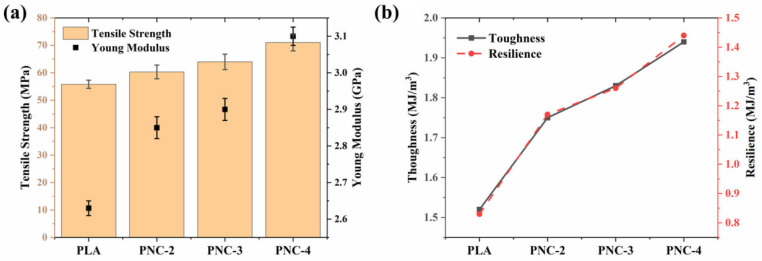
(**a**) Tensile strength and tensile modulus, and (**b**) toughness and resilience of PLA, PNC-2, PNC-3, and PNC-4 FDM 3D-printed samples.

The effect of reinforcing CNTs into the PLA matrix on the mechanical properties of FDM 3D-printed samples was evaluated. The results were compared with an FDM 3D-printed Virgin PLA specimen (Figure 7). The virgin PLA showcased an average tensile strength of 56 MPa, a tensile modulus of 2.62 GPa, and an elongation at break of 4.1%. Via the successive addition of CNTs to PLA, a notable improvement in mechanical properties was observed, as detailed in Table 5. Herein, the applied tensile force to the PNCs was effectively transferred to the CNTs, resulting in an enhancement in both the tensile strength and modulus of the 3D-printed PNC samples. Notably, the incorporation of 3 wt.% CNTs into PLA (referred to as PNC-4) resulted in a 27.24% increase in tensile strength and a 17.78% enhancement in modulus compared to virgin PLA. A similar pattern was evident in other mechanical properties, where the elongation at break, toughness, and resilience faced an increase of 73%, 27.63%, and 73.5%, respectively.

### 4.6. Electrochemical Performance and Morphology

**Figure 8:**
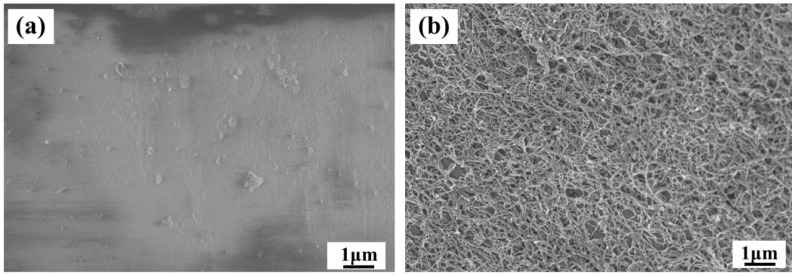
SEM micrographs of (**a**) as-printed and (**b**) electrochemical-activated FDM 3D-printed PNC-3.

**Figure 9:**
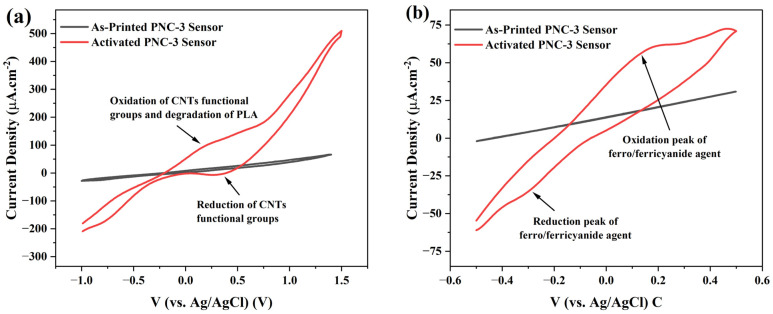
(**a**) Cyclic voltammograms obtained with 3D-printed PNC-3 sensor in 0.5 M NaOH solution before (black line) and after electrochemical activation for 250 cycles (red line). Scan rate: 50 mV/s. Step potential: 10 mV. (**b**) Cyclic voltammograms obtained in the present of ferro/ferricyanide redox couple before (black line) and after (red line) electrochemical activation of PNC-3 working electrode in NaOH 0.5 M. Scan rate: 50 mV/s. Step potential: 10 mV.

The electrochemical characteristics of a 3D-printed PNC-3 (2 wt.% of CNTs) were particularly examined to evaluate its potential applicability in electrochemical-sensing applications. To this end, the samples were electrochemically surface-activated using cyclic voltammetry in a 0.5 M NaOH electrolyte for 250 cycles and compared with as-printed samples (Figure 8 and Figure 9a). Oxidation peaks in Figure 9a (red line), subsequent to the 250-cycle voltammetry in NaOH, attributed to the formation of oxygen-containing functional groups on the CNT surface and/or electrochemical degradation of PLA, while reduction peaks can be attributed to the reduction of oxygen-containing functional groups on the CNTs, representing the electrode’s electrocatalytic activity. By contrast, the as-printed PNC-3 electrode exhibited almost no response peak, as illustrated in Figure 9a (black line), which signifies the presence of the insulating PLA layer on the as-printed surface.

Field emission scanning electron microscopy (FE-SEM) was employed to assess the morphology of the surface of the PNC-3 before and after surface activation in NaOH (Figure 8). The FE-SEM image presented in Figure 8a demonstrates a complete surface coverage of the polymer matrix, with CNTs embedded within. Figure 8b illustrates a homogeneously distributed layer of CNTs on the surface, resulting from the electrochemical activation process that effectively removed the PLA layer from the surface.

As a proof of concept, Figure 9b illustrates the cyclic voltammetry behavior of both as-printed (black line) and electro-activated (red line) 3D-printed PNC-3 in a 5 mM [Fe(CN)_6_]^4−/3−^ redox agent solution, at a 50 mV/s scan rate. The redox peaks observed are the characteristics of the reversible electron transfer reactions between ferricyanide Fe (CN)_6_^3−^ and ferrocyanide Fe(CN)_6_^4−^ species, showing the electrochemical performance of 3D-printed CNT-base PLA nanocomposite electrode and its catalytic efficiency, conductivity, and electrochemical stability [13]. The interaction between the charged ferro/ferricyanide analyte and the activated electrode surface promotes efficient electron transfer, likely due to the enhanced active sites provided by exposed CNTs on the surface via the electro-activation of the electrode. The interaction between the analyte and CNTs underscores the binding affinity of ferro/ferricyanide at the electrode–electrolyte interface, contributing to improved signal stability and detection sensitivity [50]. Notably, the activated electrode exhibited an oxidation peak with a current density of 17.6 μA.cm^−2^, highlighting the enhanced current response linked to increased analyte binding at activated sites.

To compare the outcome of this study with the open literature, Table 6 compares similar works, highlighting the measured conductivity of FDM-printed samples at different CNT concentrations within the PLA matrix. While there are some studies using the melt mixing method, which has achieved a low percolation threshold with high conductivity (e.g., 0.5 wt.% of CNTs and conductivity of 5 × 10^−7^ S.m^−1^ [26]; 1.5 wt.% CNTs and conductivity of approximately 1 × 10^−8^ S.m^−1^ [51]), our study represents an eco-friendly solution-casting approach for the first time, utilizing ethyl acetate as green solvent, to uniformly distribute CNTs inside polymer, without using a high temperature, and achieve a low percolation threshold onset of 1 wt.% of CNT (5 × 10^−10^ S.m^−1^). The only two other studies that using solution-casting methods available in the literature used toxic solvents to prepare their feedstock, dichloromethane (DCM) and chloroform [29,30]. Out of the two studies mentioned, one did not report the percolation threshold [29], while the other study investigated only adding 10 wt.% CNTs [30]. Furthermore, our study expands the scope of comprehensive investigation beyond the property evaluations (e.g., thermal, mechanical, and electrical properties) by further studying and comparing the electrochemical response of the developed 3D-printed nanocomposites, an area that has been scarcely addressed previously.

## 5. Conclusions

The current study investigates the development of a high-performance nanocomposite by incorporating CNTs into PLA biopolymer using an eco-friendly process. Notably, this approach marks the first utilization of ethyl acetate, an environmentally friendly solvent, in solution mixing to fabricate bio-nanocomposite feedstock, suitable for FDM 3D printing. Varying CNT concentrations from 0.5 to 3 wt.% allowed for a systematic investigation of CNTs’ effect on the physicochemical and mechanical properties of 3D-printed parts. For example, the addition of only 2 wt.% CNTs led to a substantial increase in electrical conductivity, from approximately 1 × 10^−12^ S.m^−1^ for virgin PLA to 8.3 × 10^−3^ S.m^−1^. The significant shift in the electrical conductivity indicates a percolation threshold at 2 wt.% CNTs, attributed to the uniform CNTs dispersion achieved through the green solution mixing. Our rheological analysis revealed an increase in complex and shear viscosity with the sequential addition of CNTs, attributed to the reinforcing effect of CNTs within the PLA matrix. Specifically, a notable rise in complex viscosity from PNC-2 to PNC-3 is indicative of a percolated network of CNTs, suggesting enhanced interaction between the CNTs and the polymer chain. Additionally, the degree of crystallinity increased with the addition of CNTs, as evidenced in both initial and subsequent heating scans. The mechanical properties of 3D-printed samples demonstrated an enhanced performance with increasing CNT content; specifically, the PNC-3 sample containing 2 wt.% CNTs exhibited a 14.5% increase in tensile strength and a 10.3% increase in modulus compared to virgin PLA. Electrochemical surface activation was further applied to improve the electrochemical response to the ferro/ferricyanide redox agent, highlighting the role of CNTs in enhancing signal stability and detection sensitivity through improved analyte binding and electron transfer. Overall, the findings underscore the potential of sustainable feedstock preparation and cost-effective 3D-printing approach for electrochemical-sensing applications. However, challenges remain, particularly in optimizing printing parameters to ensure consistent performance and reliable sensitivity comparable to conventional electrochemical sensors. Future work would focus on applying the developed PNCs’ bio-nanocomposites in electrochemical-sensing applications, such as glucose detection, which is currently under investigation by our group.

## Figures and Tables

**Table 1:** Composite formulations and code chart.

Code Chart	PLA (wt.%)	MWCNTs-COOH (wt.%)
PLA	100	0
D-PLA	100	0
PNC-1	99.5	0.5
PNC-2	99	1
PNC-3	98	2
PNC-4	97	3

D-PLA, dissolved PLA in ethyl acetate. PNC, polymeric nanocomposite.

**Table 2:** FDM 3D-printing parameters.

Printing Parameters	
Nozzle diameter	0.4 mm
Nozzle temperature	200 °C
Bed temperature	60 °C
Infill density	100%
Layer height	0.2 mm
Raster angle	45°
Print speed	10 mm/s
Flow rate	100%

**Table 3:** DSC tabulated results and calculated degree of crystallinity (%X_Cr_).

	First Heating Cycle				Second Heating Cycle			
	T_g_ (°C)	T_CC_ (°C)	T_m_ (°C)	%X_Cr_	T_g_ (°C)	T_CC_ (°C)	T_m_ (°C)	%X_Cr_
PLA	59	106	147	6.96	61	113	149	4.1
D-PLA	59	108	147 *	3.49	61	109	147 *	3
PNC-1	59	106	146	5.3	61	127	151	3.68
PNC-2	59	105	147	14.95	61	128	151	4.23
PNC-3	60	99	148	15.78	61	129	151	4.62
PNC-4	60	97	149	16.45	61	130	151	5.22

* D-PLA illustrated two distinct melting peaks, at 147 and 153 °C, for both the first and second heating rate.

**Table 4:** Zero-shear viscosity data.

	Zero-Shear Viscosity (Pa.S)	R^2^
PLA	3.03 × 10^2^	0.93
D-PLA	9.39 × 10^2^	0.96
PNC-1	1.76 × 10^5^	0.99
PNC-2	2.81 × 10^5^	0.99
PNC-3	6.46 × 10^5^	0.99
PNC-4	1.6 × 10^5^	0.99

**Table 5:** Mechanical properties of FDM 3D-printed PLA, PNC-2, PNC-3, and PNC-4.

	PLA	PNC-2	PNC-3	PNC-4
Tensile strength (MPa)	55.8 ± 1.4	60.5 ± 2.35	63.9 ± 2.8	71 ± 3.2
Tensile modulus (GPa)	2.63 ± 0.1	2.84 ± 0.05	2.9 ± 0.1	3.1 ± 0.07
Elongation at break (%)	4.1 ± 0.2	5.32 ± 0.5	6.5 ± 0.3	7.1 ± 0.2
Toughness (MJ/m^3^)	1.52 ± 0.01	1.75 ± 0.03	1.83 ± 0.01	1.94 ± 0.02
Resilience (MJ/m^3^)	0.83 ± 0.01	1.17 ± 0.02	1.26 ± 0.01	1.44 ± 0.04

**Table 6:** Comparison of FDM 3D-printed PLA:CNT composite developed in this study with the open literature.

Nanocomposite Feedstock Preparation Method	CNTs wt.% and Related Conductivity (S.m^−1^)				Percolation Threshold Onset		Ref.
	0.5	1	2	3	Wt.%	Conductivity (S.m^−1^)	
Solution casting using Ethyl acetate	1 × 10^−10^	5 × 10^−10^	8.3 × 10^−3^	5 × 10^−2^	1	5 × 10^−10^	Our Work
Melt extrusion	N/A	N/A	N/A	1 × 10^−2^	1.5	1 × 10^−8^	[51]
Melt extrusion	N/A	N/A	N/A	7.8 × 10^−4^	1.5	1.4 × 10^−8^	[25]
Melt extrusion	5 × 10^−7^	1.5 × 10^−3^	3 × 10^−3^	1	0.5	5 × 10^−7^	[26]
Mechanical mixing of PLA powder with CNTs	1 × 10^−9^	5 × 10^−5^	N/A	N/A	0.5	1 × 10^−9^	[52]
Mechanical mixing of PLA powder with CNTs	1 × 10^−9^	5 × 10^−5^	N/A	N/A	0.5	5 × 10^−5^	[53]
Solution casting using DCM	N/A	1 × 10^−4^	1 × 10^−1^	5 × 10^−1^	N/A	N/A	[29]
Solution casting using chloroform *	N/A	N/A	N/A	N/A	N/A	N/A	[30]

* Solution mixing with the aid of incorporating polycaprolactone (PCL) and poly(styrene-butadiene-styrene) (SBS) into the mixture (each 10 wt.%). Also, the only paper evaluated the sensing performance of the developed electrode.

## Data Availability

All data generated and/or analyzed during this study are included in the published article.

## References

[1] Erdem A., Yildiz E., Senturk H., Maral M. (2023). Implementation of 3D printing technologies to electrochemical and optical biosensors developed for biomedical and pharmaceutical analysis. J. Pharm. Biomed. Anal..

[2] Mohammadpour-Haratbar A., Zare Y., Rhee K.Y. (2022). Electrochemical biosensors based on polymer nanocomposites for detecting breast cancer: Recent progress and future prospects. Adv. Colloid Interface Sci..

[3] Gamal O., Eldin M.H., Refaat A.A., Hassan R.Y.A. (2024). Advances in nanocomposites-based electrochemical biosensors for the early diagnosis of breast cancer. Front. Sens..

[4] Rasal R.K., Badsha I., Shellaiah M., Subramanian K., Gayathri A., Hirad A.H., Kaliaperumal K., Devasena T. (2024). Fabrication of Curcumin-Based Electrochemical Nanosensors for the Detection of Environmental Pollutants: 1,4-Dioxane and Hydrazine. Biosensors.

[5] Durai L., Badhulika S. (2022). Current Challenges and Developments in Perovskite-Based Electrochemical Biosensors for Effective Theragnostics of Neurological Disorders. ACS Omega.

[6] Sharma A., Faber H., Khosla A., Anthopoulos T.D. (2023). 3D printed electrochemical devices for bio-chemical sensing: A review. Mater. Sci. Eng. R Rep..

[7] Osman A., Lu J. (2023). 3D printing of polymer composites to fabricate wearable sensors: A comprehensive review. Mater. Sci. Eng. R Rep..

[8] Hussan KS J., Subramaniam M.P., Kenz K.T.M., Sreeram P., Parvathi S., Sari P.S., Pullanchiyodan A., Mulhivill D.M., Raghavan P. (2024). Fabrication and challenges of 3D printed sensors for biomedical applications-Comprehensive review. Results Eng..

[9] Karimi N., Fayazfar H. (2023). Development of highly filled nickel-polymer feedstock from recycled and biodegradable resources for low-cost material extrusion additive manufacturing of metals. J. Manuf. Process.

[10] Tan L.J., Zhu W., Zhou K. (2020). Recent Progress on Polymer Materials for Additive Manufacturing. Adv. Funct. Mater..

[11] Martins P., Pereira N., Lima A.C., Garcia A., Mendes-Filipe C., Policia R., Correia V., Lanceros-Mendez S. (2023). Advances in Printing and Electronics: From Engagement to Commitment. Adv. Funct. Mater..

[12] Katseli V., Economou A., Kokkinos C. (2019). Single-step fabrication of an integrated 3D-printed device for electrochemical sensing applications. Electrochem. Commun..

[13] Sharifi J., Rizvi G., Fayazfar H. (2024). Sustainable 3D printing of enhanced carbon nanotube-based polymeric nanocomposites: Green solvent-based casting for eco-friendly electrochemical sensing applications. Int. J. Adv. Manuf. Technol..

[14] Shinyama K. (2018). Influence of Electron Beam Irradiation on Electrical Insulating Properties of PLA with Soft Resin Added. Polymers.

[15] Azizi-Lalabadi M., Jafari S.M. (2021). Bio-nanocomposites of graphene with biopolymers; fabrication, properties, and applications. Adv. Colloid Interface Sci..

[16] Shameem M.M., Sasikanth S.M., Annamalai R., Raman R.G. (2021). A brief review on polymer nanocomposites and its applications. Mater. Today Proc..

[17] Carroccio S.C., Scarfato P., Bruno E., Aprea P., Dintcheva N.T., Filippone G. (2022). Impact of nanoparticles on the environmental sustainability of polymer nanocomposites based on bioplastics or recycled plastics—A review of life-cycle assessment studies. J. Clean. Prod..

[18] Ferrier D.C., Honeychurch K.C. (2021). Carbon nanotube (CNT)-based biosensors. Biosensors.

[19] Karimi F., Karimi-Maleh H., Rouhi J., Zare N., Karaman C., Baghayeri M., Fu L., Rostamnia S., Dragoi E.N., Ayati A. (2023). Revolutionizing cancer monitoring with carbon-based electrochemical biosensors. Environ. Res..

[20] Ma P.C., Siddiqui N.A., Marom G., Kim J.K. (2010). Dispersion and functionalization of carbon nanotubes for polymer-based nanocomposites: A review. Compos. Part A Appl. Sci. Manuf..

[21] Soni S.K., Thomas B., Kar V.R. (2020). A Comprehensive Review on CNTs and CNT-Reinforced Composites: Syntheses, Characteristics and Applications. Mater. Today Commun..

[22] Mi D., Zhao Z., Bai H. (2023). Improved Yield and Electrical Properties of Poly(Lactic Acid)/Carbon Nanotube Composites by Shear and Anneal. Materials.

[23] Khan T., Irfan M., Ali M., Dong Y., Ramakrishna S., Umer R. (2021). Insights to low electrical percolation thresholds of carbon-based polypropylene nanocomposites. Carbon.

[24] Ong Y.T., Tan S.H. (2019). Carbon nanotube-based biodegradable polymeric nanocomposites: 3Rs (Reduce, Reuse, and Recycle) in the design. Handb. Ecomater..

[25] Ivanov E., Kotsilkova R., Xia H., Chen Y., Donato R.K., Donato K., Godoy A.P., Di Maio R., Silvestre C., Cimmino S. (2019). PLA/Graphene/MWCNT Composites with Improved Electrical and Thermal Properties Suitable for FDM 3D Printing Applications. Appl. Sci..

[26] Mora A., Verma P., Kumar S. (2020). Electrical conductivity of CNT/polymer composites: 3D printing, measurements and modeling. Compos. Part B Eng..

[27] Ke K., Wang Y., Liu X.-Q., Cao J., Luo Y., Yang W., Xie B.-H., Yang M.-B. (2012). A comparison of melt and solution mixing on the dispersion of carbon nanotubes in a poly(vinylidene fluoride) matrix. Compos. Part B Eng..

[28] Spinelli G., Lamberti P., Tucci V., Kotsilkova R., Tabakova S., Ivanova R., Angelova P., Angelov V., Ivanov E., Di Maio R. (2018). Morphological, Rheological and Electromagnetic Properties of Nanocarbon/Poly(lactic) Acid for 3D Printing: Solution Blending vs. Melt Mixing. Materials.

[29] Kim H.-G., Hajra S., Oh D., Kim N., Kim H.J. (2021). Additive manufacturing of high-performance carbon-composites: An integrated multi-axis pressure and temperature monitoring sensor. Compos. Part B Eng..

[30] Junpha J., Wisitsoraat A., Prathumwan R., Chaengsawang W., Khomungkhun K., Subannajui K. (2020). Electronic tongue and cyclic voltammetric sensors based on carbon nanotube/polylactic composites fabricated by fused deposition modelling 3D printing. Mater. Sci. Eng. C.

[31] Torkelson T.R., Oyen F., Rowe V.K. (1976). The toxicity of chloroform as determined by single and repeated exposure of laboratory animals. Am. Ind. Hyg. Assoc. J..

[32] Schlosser P.M., Bale A.S., Gibbons C.F., Wilkins A., Cooper G.S. (2014). Human Health Effects of Dichloromethane: Key Findings and Scientific Issues. Environ. Health Perspect..

[33] Han W., Rao D., Gao H., Yang X., Fan H., Li C., Dong L., Meng H. (2022). Green-solvent-processable biodegradable poly(lactic acid) nanofibrous membranes with bead-on-string structure for effective air filtration: “Kill two birds with one stone”. Nano Energy.

[34] Vergaelen M., Verbraeken B., Van Guyse J.F.R., Podevyn A., Tigrine A., de la Rosa V.R., Monnery B.D., Hoogenboom R. (2020). Ethyl acetate as solvent for the synthesis of poly(2-ethyl-2-oxazoline). Green Chem..

[35] De Bortoli L., de Farias R., Mezalira D., Schabbach L., Fredel M. (2022). Functionalized carbon nanotubes for 3D-printed PLA-nanocomposites: Effects on thermal and mechanical properties. Mater. Today Commun..

[36] Jubinville D., Sharifi J., Mekonnen T.H., Fayazfar H. (2023). A Comparative Study of the Physico-Mechanical Properties of Material Extrusion 3D-Printed and Injection Molded Wood-Polymeric Biocomposites. J. Polym. Environ..

[37] Dealy J.M., Wissbrun K.F. (1999). Theory and Applications. Melt Rheology and Its Role in Plastics Processing.

[38] (2022). Standard Test Method for Tensile Properties of Plastics.

[39] Rocha D.P., Rocha R.G., Castro S.V.F., Trindade M.A.G., Munoz R.A.A., Richter E.M., Angnes L. (2022). Posttreatment of 3D-printed surfaces for electrochemical applications: A critical review on proposed protocols. Electrochem. Sci. Adv..

[40] Park S.G., Abdal-Hay A., Lim J.K. (2015). Biodegradable poly(lactic acid)/multiwalled carbon nanotube nanocomposite fabrication using casting and hot press techniques. Arch. Metall. Mater..

[41] Zhang C., Lan Q., Zhai T., Nie S., Luo J., Yan W. (2018). Melt crystallization behavior and crystalline morphology of Polylactide/Poly(ε-caprolactone) blends compatibilized by lactide-caprolactone copolymer. Polymers.

[42] Petrovics N., Kirchkeszner C., Patkó A., Tábi T., Magyar N., Székely I.K., Szabó B.S., Nyiri Z., Eke Z. (2023). Effect of crystallinity on the migration of plastic additives from polylactic acid-based food contact plastics. Food Packag. Shelf Life.

[43] Papadopoulos L., Klonos P.A., Terzopoulou Z., Psochia E., Sanusi O.M., Hocine N.A., Benelfellah A., Giliopoulos D., Triantafyllidis K., Kyritsis A. (2021). Comparative study of crystallization, semicrystalline morphology, and molecular mobility in nanocomposites based on polylactide and various inclusions at low filler loadings. Polymer.

[44] Li Y., Yin D., Liu W., Zhou H., Zhang Y., Wang X. (2020). Fabrication of biodegradable poly (lactic acid)/carbon nanotube nanocomposite foams: Significant improvement on rheological property and foamability. Int. J. Biol. Macromol..

[45] Ali F., Ishfaq N., Said A., Nawaz Z., Ali Z., Ali N., Afzal A., Bilal M. (2021). Fabrication, characterization, morphological and thermal investigations of functionalized multi-walled carbon nanotubes reinforced epoxy nanocomposites. Prog. Org. Coat..

[46] Jubinville D., Sharifi J., Fayazfar H., Mekonnen T.H. (2023). Hemp hurd filled PLA-PBAT blend biocomposites compatible with additive manufacturing processes: Fabrication, rheology, and material property investigations. Polym. Compos..

[47] Xu Z., Niu Y., Yang L., Xie W., Li H., Gan Z., Wang Z. (2010). Morphology, rheology and crystallization behavior of polylactide composites prepared through addition of five-armed star polylactide grafted multiwalled carbon nanotubes. Polymer.

[48] Xue Q., Sun J. (2016). Electrical conductivity and percolation behavior of polymer nanocomposites. Polymer Nanocomposites: Electrical and Thermal Properties.

[49] Al-Saleh M.H., Sundararaj U. (2009). A review of vapor grown carbon nanofiber/polymer conductive composites. Carbon.

[50] Poghossian A., Weil M., Cherstvy A.G., Schöning M.J. (2013). Electrical monitoring of polyelectrolyte multilayer formation by means of capacitive field-effect devices. Anal. Bioanal. Chem..

[51] Lamberti P., Spinelli G., Kuzhir P.P., Guadagno L., Naddeo C., Romano V., Kotsilkova R., Angelova P., Georgiev V. (2018). Evaluation of thermal and electrical conductivity of carbon-based PLA nanocomposites for 3D printing. AIP Conf. Proc..

[52] Vidakis N., Petousis M., Kourinou M., Velidakis E., Mountakis N., Fischer-Griffiths P.E., Grammatikos S., Tzounis L. (2021). Additive manufacturing of multifunctional polylactic acid (PLA)—Multiwalled carbon nanotubes (MWCNTs) nanocomposites. Nanocomposites.

[53] Petousis M., Ninikas K., Vidakis N., Mountakis N., Kechagias J.D. (2023). Multifunctional PLA/CNTs nanocomposites hybrid 3D printing integrating material extrusion and CO_2_ laser cutting. J. Manuf. Process.

